# Suitability of Dried Blood Spots for Accelerating Veterinary Biobank Collections and Identifying Metabolomics Biomarkers With Minimal Resources

**DOI:** 10.3389/fvets.2022.887163

**Published:** 2022-06-22

**Authors:** David Allaway, Janet E. Alexander, Laura J. Carvell-Miller, Rhiannon M. Reynolds, Catherine L. Winder, Ralf J. M. Weber, Gavin R. Lloyd, Andrew D. Southam, Warwick B. Dunn

**Affiliations:** ^1^WALTHAM Petcare Science Institute, Freeby Lane, Waltham-on-the-Wolds, Melton Mowbray, United Kingdom; ^2^School of Biosciences and Phenome Centre Birmingham, University of Birmingham, Birmingham, United Kingdom; ^3^Department of Biochemistry and Systems Biology, Institute of Systems, Molecular, and Integrative Biology, University of Liverpool, Liverpool, United Kingdom; ^4^Institute of Metabolism and Systems Research, University of Birmingham, Birmingham, United Kingdom

**Keywords:** dried blood spots (DBSs), biobank, metabolomics, biomarker, dog, cat

## Abstract

Biomarker discovery using biobank samples collected from veterinary clinics would deliver insights into the diverse population of pets and accelerate diagnostic development. The acquisition, preparation, processing, and storage of biofluid samples in sufficient volumes and at a quality suitable for later analysis with most suitable discovery methods remain challenging. Metabolomics analysis is a valuable approach to detect health/disease phenotypes. Pre-processing changes during preparation of plasma/serum samples may induce variability that may be overcome using dried blood spots (DBSs). We report a proof of principle study by metabolite fingerprinting applying UHPLC-MS of plasma and DBSs acquired from healthy adult dogs and cats (age range 1–9 years), representing each of 4 dog breeds (Labrador retriever, Beagle, Petit Basset Griffon Vendeen, and Norfolk terrier) and the British domestic shorthair cat (n = 10 per group). Blood samples (20 and 40 μL) for DBSs were loaded onto filter paper, air-dried at room temperature (3 h), and sealed and stored (4°C for ~72 h) prior to storage at −80°C. Plasma from the same blood draw (250 μL) was prepared and stored at −80°C within 1 h of sampling. Metabolite fingerprinting of the DBSs and plasma produced similar numbers of metabolite features that had similar abilities to discriminate between biological classes and correctly assign blinded samples. These provide evidence that DBSs, sampled in a manner amenable to application in in-clinic/in-field processing, are a suitable sample for biomarker discovery using UHPLC-MS metabolomics. Further, given appropriate owner consent, the volumes tested (20–40 μL) make the acquisition of remnant blood from blood samples drawn for other reasons available for biobanking and other research activities. Together, this makes possible large-scale biobanking of veterinary samples, gaining sufficient material sooner and enabling quicker identification of biomarkers of interest.

## Introduction

The availability of earlier and more specific diagnostic tests would support clinicians in diagnosing and treating patients. An initial challenge in the development of diagnostic tests is access to a sufficient number of relevant and high-quality samples from multiple different geographical locations to provide robust statistical power for biomarker discovery. Blood (along with urine) is a relatively common biological sample taken for routine clinical analysis and, as a systemic biofluid, is suitable for identification of biomarkers related to the function of most body organs and tissues. However, establishing routine plasma/serum-based biobanks of sufficient quality across primary veterinary clinics has some logistical challenges, especially related to access to laboratory equipment for processing of whole blood from its plasma or serum fractions into aliquots and quickly freezing these at ultralow temperatures. Alternative methods that can access and stabilize small blood volumes with minimal equipment and time resources would be advantageous. The use of blood obtained by needle prick, blotted and dried on filter paper (dried blood spots, DBS) in the diagnosis of human inborn errors soon after birth (Guthrie card) (1, 2) reflects the minimally invasive approach and relative ease of sampling. The approach has been developed further for pharmacokinetics and drug trials (therapeutics and toxicology) (3–5) where samples can be collected, applied to a filter, dried, stored (in some cases without the need for freezing), and transported at a low cost and with minimal resources. Furthermore, human DBS samples have been evaluated as a biosample for biomarker discovery by screening biobanked samples using several -omics platforms (genomics, transcriptomics, proteomics, and metabolomics) (6).

Key logistical advantages of DBS over plasma/serum include (1) the ability to use a minimal whole blood sample (~20–50 μL *cf*. 1–2 ml for plasma/serum collection); (2) minimal preparation time/equipment required for sampling and sample processing; (3) no need for a trained phlebotomist using a hypodermic needle; (4) the ability to transport chilled/at ambient temperature results in much lower transport costs; (5) once dried, sample stability for 1–2 weeks at ambient temperature. These advantages make the collection, preparation, and storage of whole blood for biobanking far less challenging than the standard samples (plasma/serum) and more suitable for implementation in veterinary clinics or even pet owners' homes. Such an approach would enable more samples to be acquired more quickly to be available for biomarker discovery and validation.

Metabolomics, the global measurement of metabolites in a biological sample, is a particularly valuable approach to detect health/disease phenotypes, as metabolites represent the interface of intrinsic genetics, gene expression, and protein activities of individuals, as well as physiology (life stage, gender, and chronobiology) and the environment (diet, gut microbiome, drugs, and exposome) (7, 8). Also, unlike genomes, transcripts, and proteins, metabolites are universally identical across species (i.e., glucose is always glucose), enabling direct comparisons across different biological classes. Metabolites are also relatively easy targets for developing diagnostic assays, and there are advantages when looking to validate biomarkers in other species with similar pathologies. One concern for metabolomics analysis from whole blood collected in clinics is the potential for *ex vivo* metabolism (9). One approach to investigate this is using whole blood incubated with stable isotope-labeled U^13^C-glucose immediately prior to storage as liquid or DBS (10). The authors concluded that DBSs lower the risk of *ex vivo* metabolism compared to liquid blood under the same conditions. While they did not compare DBSs to plasma stored under appropriate conditions (at 4°C), these observations indicate that DBSs may provide a practical and valuable source of metabolites for biomarker discovery. Application of metabolomics for disease biomarker discovery using DBSs has been reported (i.e., for polyp and breast cancer) (11, 12). Other studies have applied metabolomics to investigate the stability of a wide range of metabolite and lipid concentrations and have demonstrated that DBS samples are stable for 1–2 weeks post collection at ambient temperatures (13–15).

Detailed literature reviews describe the use of DBSs in medical and research applications [i.e., (16), best practices (17), and applications to measure specific analytes (18–20)]. Plasma metabolomics studies have reported metabolic differences related to breed and size differences in dogs (21, 22), diet effects in cats and dogs (23), and the impact of neutering and life stage in cats (23).

The purpose of this proof of principle study was to determine whether metabolite fingerprinting analysis of DBSs, processed in a way similar to that which could be achieved under normal veterinary conditions, could provide reliable and informative metabolomics data able to discriminate between biological classes in companion animals. To identify the impact of sample volume variability on discrimination, two DBS volumes from each sample were compared (20 and 40 μL). To provide a range of biological diversity and gauge the extent to which DBSs can discriminate using a small set of samples, differences based on species (dog and cat), dog breed and size (small, medium, and large) were chosen and compared to the discrimination provided by the plasma collected and processed under optimal conditions.

## Methods

### Study Design

Fifty animals (minimum of 10 per class) housed at the WALTHAM Petcare Science Institute according to the code of practice that sets out the standards of care and accommodation of animals required by the Animals (Scientific) Procedures Act 1986 took part in the study. During each animal's biannual health check, an additional blood sample (1 ml) was drawn to provide a plasma sample (250 μL) and five DBS cards (each card having 5 × 20 μL spots). The plasma was prepared using laboratory best practice, while DBS cards were treated under credible veterinary clinic conditions (4°C for 72 h) prior to long-term storage at −80°C. The study was approved by the WALTHAM Animal Welfare and Ethical Review Body.

### Animals

Healthy adult dogs and cats (age range 1–9 years), representing each of 4 dog breeds [Labrador retriever (large breed), Beagle (medium breed), Petit Basset Griffon Vendeen (PBGV; medium breed), and Norfolk terrier (small breed)] and the British domestic shorthair cat on their standard maintenance diet were recruited for the study. Ten individuals per group were balanced for gender (1:1 male:female). On two occasions, plasma samples were not prepared within the timelines required, and two additional animals had a blood sample taken to provide sufficient samples. The DBS samples from the two void animals were provided as “blinded” samples to analysts and statisticians, allowing for an *ad hoc* test of the ability to assign unknown samples to the correct classes (species and, if required, breed size and breed).

### Blood Sampling and Sample Preparation

A fasted (> 12 h) blood sample (1 ml) from the jugular venipuncture site was placed in a lithium heparin tube and kept on ice until it was received in the lab (time to lab was < 30 min) where the plasma (250 μL) was prepared by centrifugation (time from sampling to −80°C was < 120 min). For DBSs, blood (0.5 ml) was drawn into a multi-pipettor, and aliquots (20 μL) were applied to individual sites on Whatman™903 Protein Saver cards (5 spots per card, 5 cards) (product number: 11962089). The blood spots were air-dried at room temperature for 3 h prior to being individually sealed in Whatman™903 Foil-Barrier Sample Bags (product number: 11984265) with a dessicated Whatman™ FTA™ Dessicant Packet (Product Number: 10111442). The cards were stored at 4°C for ~72 h prior to storage at −80°C. A set of DBS cards and the plasma sample were sent on dry ice to the University of Birmingham for metabolite fingerprinting analysis.

### Sample Extraction

DBS samples: the card containing one DBS (20 μL) was completely excised from the card using scissors, cut into four similar sized pieces, and placed in a 1.5-ml Eppendorf tube. Water (20 μL) was added, followed by addition of 250 μL methanol/acetonitrile/isopropanol [1/1/1 (v/v)] and vortex mixing for 20 min at room temperature before centrifugation (21,000 *g*, 4°C, 15 min). Two aliquots of the supernatant (70 μL) (for HILIC and lipid analyses) were transferred into 1.5-ml Eppendorf tubes, dried [SpeedVac (Savant SPD111V); Thermo Fisher Scientific, Paisley, United Kingdom), and vapor trapped (RVT5105230; Thermo Fisher Scientific, Paisley, United Kingdom)]. Additional aliquots (70 μL) from all biological sample extracts were pooled to generate a pooled QC sample and after vortex mixing, the aliquots (70 μL) were transferred to separate Eppendorf tubes and dried. The process for 40 μL DBS samples was similar with two 20 μL spots being excised and the volumes of solvents doubled with 140 μL aliquots transferred to 1.5 ml Eppendorf tubes for analysis/generation of QC samples. Two extraction blank solutions were prepared by cutting a similar sized disc of card as excised for DBS sample extraction but with no dried blood present and extracting as described above for the DBS samples. The samples were extracted prior to the day of analysis, stored and dried at −80°C, and resuspended on the first day of analysis. Preparation order for the samples was randomized using the RAND() function in Microsoft Excel. Blank, QC, and biological sample (plasma and 20 μL DBS) extracts to be analyzed in a HILIC assay were resuspended in 60 μL of water/methanol/acetonitrile [4/6/6 (v/v)]. Blank, QC, and biological sample extracts to be analyzed in a lipid assay were resuspended in 60 μL of water/isopropanol [1/3 (v/v)]; 40 μL DBS extracts for the HILIC assay were resuspended in 80 μL of water/methanol/acetonitrile [4/6/6 (v/v)] and for the lipid assay were resuspended in 80 μL of water/isopropanol [1/3 (v/v)].

Plasma samples: the plasma samples were thawed on ice. For the HILIC assay, plasma (40 μL) and 1/1 acetonitrile/water (v/v) (120 μL) were mixed by vortex (120 s) and centrifuged (21,000 *g*, 20 min, 4°C). For lipid assays, plasma (40 μL) and isopropanol (120 μL) were mixed by vortex (120 s) and centrifuged (21,000 g, 20 min, 4°C). The samples were extracted on the first day of analysis and were not dried and resuspended. For each assay separately, a pooled QC sample was prepared by pooling and mixing aliquots (40 μL) of each biological sample followed by transfer of 40 μL aliquots into separate Eppendorf tubes, and extraction was carried out as above. Two blank extract solutions were prepared by performing the extraction protocol with no plasma present. Note that due to the final dilution volumes differing for plasma and DBS samples, no direct comparison was made between the samples. The 40-μL plasma samples were the most diluted and the 20-μL DBS samples were the most concentrated, with the 40-μL DBS samples between the other two samples.

### Ultra High Performance Liquid Chromatography-Mass Spectrometry and Raw Data Processing

In brief, all the samples were analyzed by applying two UHPLC–MS methods in positive and negative ion modes separately using a DionexUltiMate 3000 UHPLC system coupled with a heated electrospray Q Exactive Focus mass spectrometer (Thermo Fisher Scientific). The HILIC assays used an Accucore150-Amide-HILIC column (100 mm × 2.1 mm, 2.6 μm; Thermo Fisher Scientific), and the lipid assays used a reversed-phase Hypersil GOLD C_18_ column (100 × 2.1 mm, 1.9 μm; Thermo Fisher Scientific). Analysis order for the samples was randomized, and all the samples were analyzed in a single analytical batch. Ten QC samples were injected at the start of each assay/ion mode to condition the analytical system and were then injected after every 6th biological sample, with two QC samples being analyzed at the end of each batch. The blank sample was analyzed as the 5th and as the final injection of the batch. Each assay is fully described in Supplementary Materials (Supplementary File A). Raw data processing is fully described in the supplementary information (Supplementary File B).

Data quality assessment and filtering: the data matrix constructed by raw data processing was filtered based on the data collected for QC and blank samples (24). Metabolite features were retained in the data matrix if they were: present in >90% of all the QC samples, had a peak area relative standard deviation (RSD) <30% across all the QC samples (QC11 onwards), had an extract blank/mean QC area ratio of <5%, and were present in >50% of the biological samples.

Metabolite annotation: putative metabolite annotation was performed applying the Python package BEAMSpy [RT diff = 5 s, Pearson correlation >0.70; *m/z* values of all experimentally observed peaks were searched against LIPIDMAPS (25) and all matches within 5-ppm mass error tolerance were reported]. To generate more robust compound annotations using MS/MS data, QC sample UHPLC-MS/MS data were matched to MS/MS databases using the LipidSearch software (lipid assay metabolite annotation; version 4.2.18, Thermo Fisher Scientific). LipidSearch features within the UHPLC-MS/MS data were searched against the entire *in-silico* HCD MS/MS database with 5-ppm mass error. Only annotations graded A-C were retained (grade A, all fatty acyl chains and class were completely identified; grade B, some fatty acyl chains and the class were identified; grade C, either the lipid class or some fatty acyls were identified). LipidSearch annotations were aligned to the XCMS outputs using the R programming language (https://www.R-project.org), applying a 5-ppm mass error for the MS1 data and a 5-s retention time tolerance window. All lipids described are reported to level 2 or 3 as defined by the Metabolomics Standards Initiative (26).

### Statistical Analysis

The statistical analyses carried out using MetaboAnalyst (v5) (27) included multivariate unsupervised principal components analysis (PCA) and one-way ANOVA. Data treatment was applied as defined previously (28). The PCA analyses applied missing value imputation (kNN (feature-wise), but there was no data filtering, normalization by sum, log transformation, or Pareto scaling. Classes were represented as different colors, and a 95% confidence ellipse was included for each class and for QC samples. A univariate one-way ANOVA with Tukey's HSD *post hoc* analysis was conducted to compare the breeds, and heatmap construction was applied with no missing value imputation, data filtering, normalization by sum, transformation, or scaling. The critical *p*-value for one-way-ANOVA was *p* < 0.005 after correction for multiple testing [Benjami-Hochberg method, BH (29)], and for *post hoc* analysis, it was *p* > 0.05 with no correction for multiple testing. Only metabolites that were statistically significant in the one-way ANOVA were further assessed by conducting a *post hoc* analysis. For heatmap construction, the most statistically significant metabolite features for each *post hoc* pairwise analysis were collated, metabolite features that were not annotated were removed, and if multiple features represented the same annotated metabolite, only the most statistically significant metabolite feature was retained.

Further univariate and multivariate analyses were conducted in R version 4.0.4. To visualize if a supervised method could separate the samples by breed and to predict the blinded DBS samples, PLS-DA was also conducted but only on dog data. The PLS-DA was analyzed after applying missing value imputation (kNN (feature-wise), no data filtering, normalization by sum, log transformation and Pareto scaling on the data. First, the number of components was estimated by assessing the error rates of different numbers of components. The number of optimal components was then used to evaluate the model by five-fold cross validation repeated 20 times. The first two components were plotted with 95% confidence ellipses for each group. The PLS-DA algorithm was then used to predict the breeds of the two blind samples. Each breed was treated as a dummy variable, and the predicted values from the final component were inspected, with the predicted breed being the group with the greatest predicted value.

## Results

The objective was to determine if the DBS samples collected, prepared, and stored under conditions reasonable for blood sampling in veterinary clinics (DBS stored at 4°C for 3 days prior to storage in a central location at −80°C) could provide metabolite fingerprinting data able to discriminate biological classes, tested at different levels of complexity. The study also investigated whether remnant blood volume (20 vs. 40 μL) impacted on the ability to detect metabolites and to discriminate samples based on phenotype, and included a comparison to plasma. The phenotype classes for discrimination were “species” (cat vs. dog), “dog size” (small-, medium-, and large-sized), and “breed” (4 dog breeds), with increasing difficulty to discriminate the subsequent class structures, i.e., cats and dogs were easiest to differentiate, and the four dog breeds were the most difficult to discriminate (21).

Four assays (HILIC and lipid in positive and negative ion modes) were used with 6.9–15.2% (median = 7.5%) missing values present in each dataset excluding the QC samples. An impact of sample injection order was identified for plasma samples in the HILIC negative data that were included in the first 23 injections for this assay. The data for QC and individual biological samples affected by this were removed from the dataset, reducing the number of individuals by 5, 4, 3, 2, and 2 for Norfolk Terrier, PBGV, DSH, Beagle, and Labrador retriever, respectively, but for the HILIC negative plasma dataset only. The number of metabolite features meeting the high quality criteria demonstrated that the DBSs (40 μL) had a similar number of metabolite features to plasma (40 μL) (Table 1), with ~15% fewer features observed for DBSs (20 μL) across all the four assays.

**Table 1:** Number of metabolite features meeting QC criteria, number and proportion reported as having overall significant variation between breeds by one-way ANOVA (No. features row) and number of metabolite features reported as being statistically significant in Tukey *post hoc* analyses (pairwise breed comparisons, *p* < 0.05).

	**HILIC +**			**HILIC—**			**LIPIDS +**			**LIPIDS—**		
	**DBS (20)**	**DBS (40)**	**Plasma**	**DBS (20)**	**DBS (40)**	**Plasma** ^**a**^	**DBS (20)**	**DBS (40)**	**Plasma**	**DBS (20)**	**DBS (40)**	**Plasma**
Total no. features	3,335	3,813	3,738	973	1,277	1,276	5,013	6,180	5,817	3,590	4,018	4,166
No. of significant	424	524	464	78	98	38	407	975	686	580	946	746
Percentage	12.71%	13.74%	12.41%	8.02%	7.67%	2.98%	8.12%	15.78%	11.79%	16.16%	23.54%	17.91%
Norfolk—Labrador	361	423	390	66	83	27	353	781	530	473	727	595
Norfolk—PBGV	244	288	327	46	72	28	344	848	520	467	773	502
Norfolk—Beagle	280	347	321	61	78	27	337	814	476	402	718	443
Labrador—PBGV	177	225	154	30	33	14	94	269	207	172	280	338
Labrador—Beagle	167	213	171	23	27	18	50	114	216	175	265	339
PBGV—Beagle	78	82	63	16	13	6	70	143	117	129	161	110

a *Number of representative samples reduced by 5, 4, 3, 2, and 2 for Norfolk Terriers, Petit Basset Griffon Vendeen (PBGV), DSH, Beagle, and Labrador retriever for HILIC negative plasma samples. The critical p-value for one-way-ANOVA was p > 0.005 after correction for multiple testing*.

### Multivariate Analyses Indicated That DBS Was Similar to Plasma in the Ability to Discriminate Biological Classes

**Figure 1:**
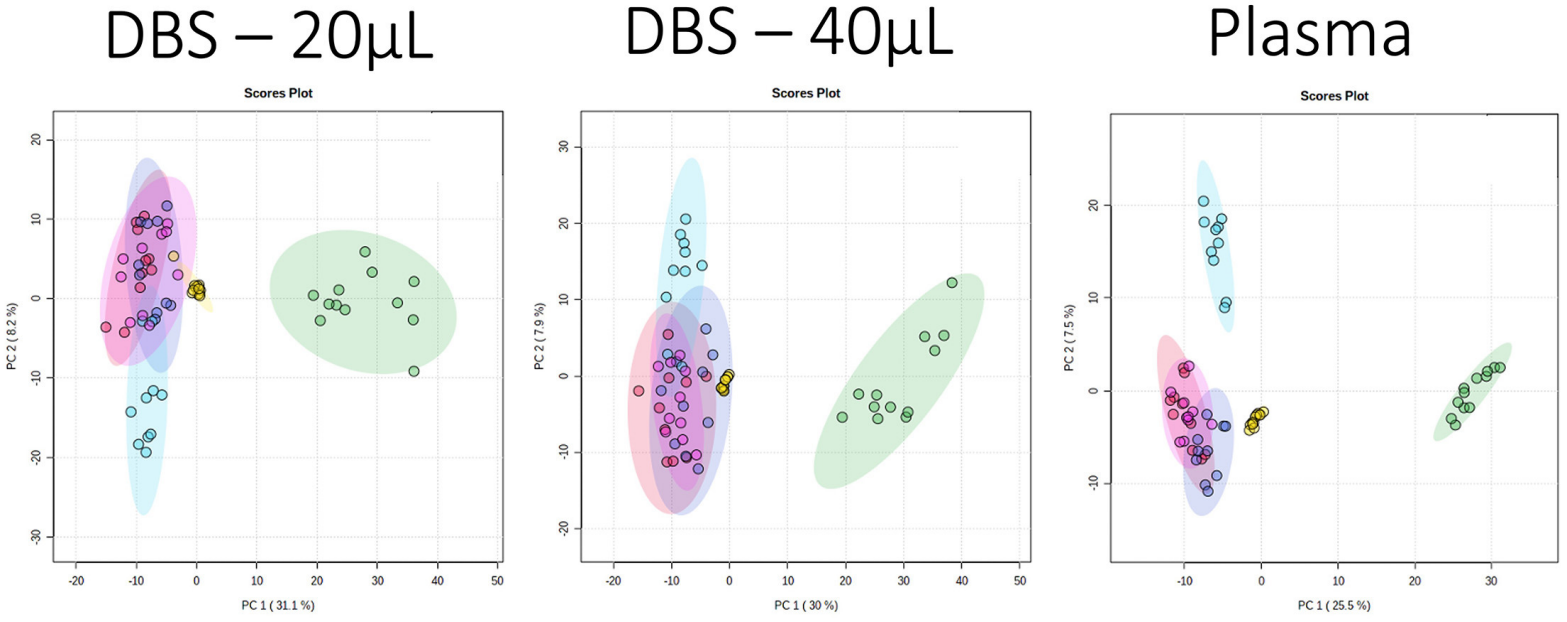
Principal component analysis (PCA) scores plots (PC1 vs. PC2) for data collected for three sample types applying the HILIC positive ion mode assay. The sample classes are pooled QC: yellow; domestic short hair cat: green, Norfolk terrier: light blue, Labrador retriever: dark blue, Beagle: red, and Petit Basset Griffon Vendeen: pink. The PC1 and PC2 variances were 31.1 and 8.2% (dried blood spot, DBS, 20 μL), 30 and 7.9% (DBS 40 μL), 25.5 and 7.5% (plasma).

PCA is an unsupervised multivariate tool that visualizes the variance and similarity of samples in a data set, two samples that are close together in a PCA score plot are defined as more similar as two samples further apart. It is often used in metabolomics to identify outliers, but if data in samples are different across a broad spectrum of the dataset, it is also possible to visualize differences between sample classes through the presence of different sample clusters. A PCA of all the samples demonstrated that the variance described in the first two components were similar between DBS and plasma, with PC1 also highlighting the difference between species (cat and dog) in all the 4 assays and all the 3 sample types (Figure 1; Supplementary File 3). The QC data also provided evidence of low analytical variability, as demonstrated by close clustering of the QC samples. Additionally, PCA plots of polar metabolites (HILIC assays) demonstrated evidence that the Norfolk terrier (small breed) partitioned from the other dog breeds in PC2. It is of note that the HILIC positive data showed some partitioning between Labrador retriever and the medium sized breeds in PC2, indicating that similar “breed size”-related data dominate variance in both DBS samples. Variance in the lipid data sets was less able to discriminate between breeds, but DBS was able to separate Norfolk terriers in lipid negative data while plasma could not. This ability of the DBS data to separate breeds may reflect breed differences in cellular metabolites not observed in plasma, such as membrane lipids.

**Figure 2:**
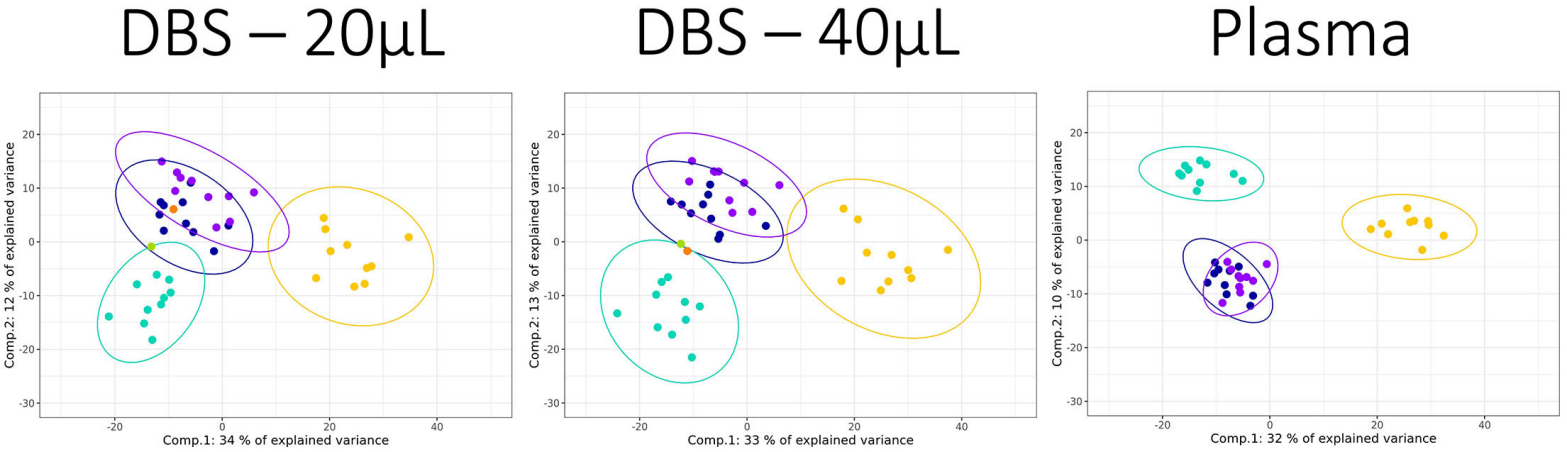
PLS-DAscores plots for dog breed data collected for three sample types applying the HILIC positive ion mode assay. The sample classes were Norfolk terrier: yellow, Labrador retriever: turquoise, Beagle: dark blue, and Petit Basset Griffon Vendeen: purple. The two blinded DBS samples, blind sample 1: orange and blind sample 2: green, were overlaid on the relevant DBS plots. The 95% confidence ellipses were plotted for each breed class. Components 1 and 2 variances were 34 and 12% (DBS 20 μL), 33 and 13% (DBS 40 μL), and 32 and 10% (plasma).

PLS-DA visualization is a supervised multivariate tool that maximizes differences in the dataset between classes while minimizing differences within class, and is often conducted in metabolomics analysis to visualize variance associated with class differences. Using dog samples with breed as a classifier, all the 3 sample types were similarly able to discriminate dogs at the “breed size” level, with good separation between the small and large dog breeds (Figure 2; Supplementary File 4). Using this supervised multivariate approach, none of the samples was able to separate the two medium-sized dog breeds, supporting the view that DBS and plasma are similar in their utility to separate these biologically complex samples. It should be noted that the two DBS samples did provide similar outputs (both % variance explained and distribution of classes across the plot), suggesting that the loss in data due to the lower volume used did not have a large impact on the discriminatory ability.

### Univariate Analysis Indicated That DBS Was Similar to Plasma in Its Ability to Discriminate Biological Classes

Often, the purpose of metabolite fingerprinting is to identify a limited number of biomarkers able to discriminate between classes of interest (e.g., healthy vs. diseased). A univariate analysis was carried out to assess whether the DBS samples could identify individual metabolites that were significantly different between classes, as were reported in plasma.

The number of statistically significant metabolite features showing differences between breeds (the most challenging class separation for multivariate analyses) using a very stringent criterion (one-way ANOVA (*p* ≤ 0.005, correction for multiple testing using the BH method) is reported in Table 1, along with the percentage in relation to the total number of metabolite features detected. Comparing the sample types, both DBS volumes and plasma had a very similar potential to provide biomarkers (both in terms of total number of features and proportion meeting the strict criteria). While the purpose of this study was only to determine the applicability of DBS as a sample for metabolomic analysis and not to identify breed-specific differences, based on the number of metabolite features that significantly differ in pair-wise comparisons (Table 1), the small Norfolk Terrier was consistently more different from the two medium-sized breeds than the larger Labrador retriever breed. Similarly, as expected from the multivariate analysis, the two medium-sized breeds had the fewest differences in both DBS and plasma. The data also demonstrated that a greater proportion of features in the lipid-based analysis was discriminatory.

**Figure 3:**
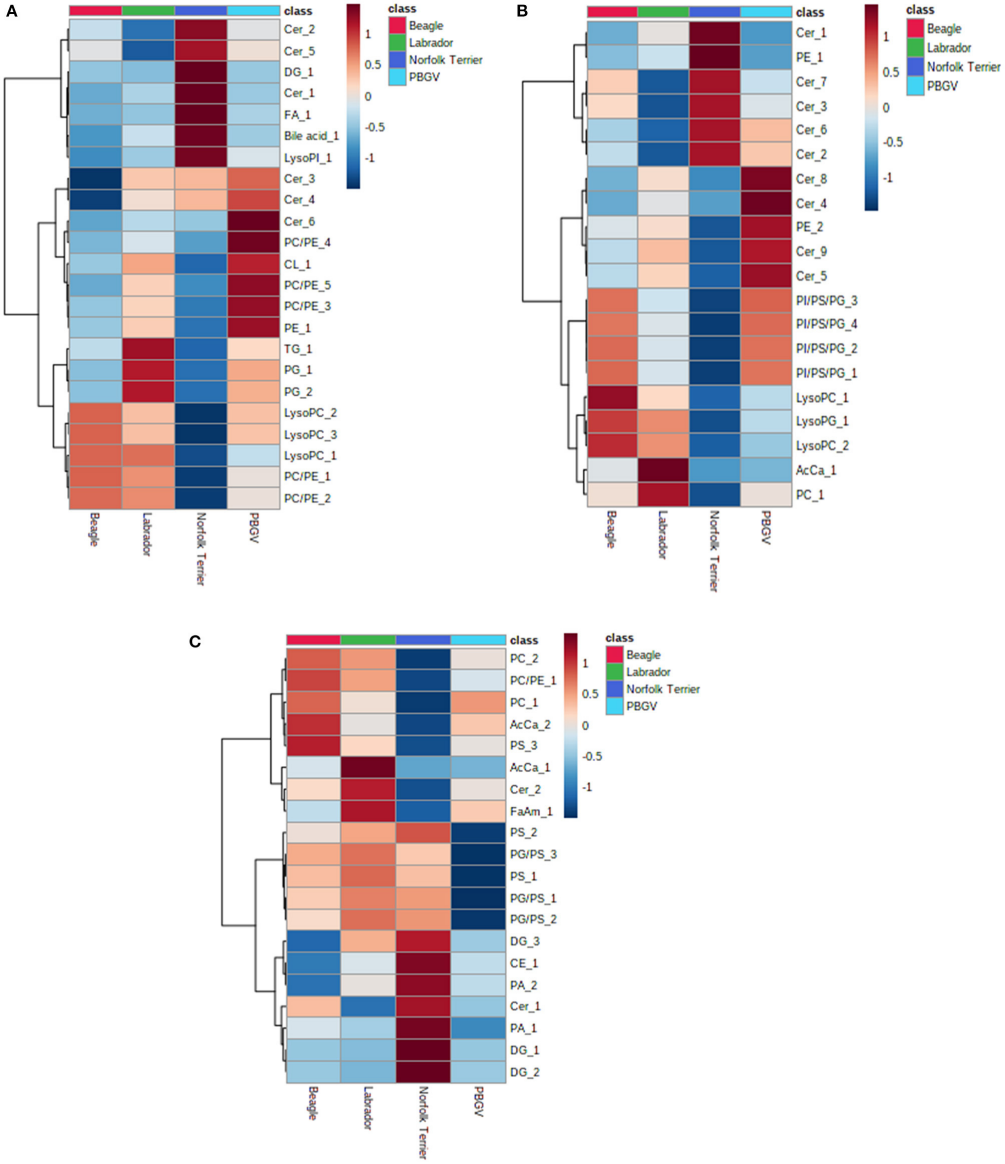
Heatmap visualization of a small data set showing breed discriminatory ability collected using the lipid positive assay for **(A)** 20 μL DBS samples, **(B)** 40 μL DBS samples, and **(C)** 40 μL plasma samples. Each breed average is colored differently (Beagle, red; Labrador retriever, green; Norfolk Terrier, dark blue; Petit Basset Griffon Vendeen, light blue). Each heatmap defines a small number of metabolites, and the breed with darkest red indicates the highest relative concentrations, darkest blue indicates the lowest concentrations, and the spectrum of blue and red in between represents intermediate concentrations. AcCa, acyl carnitine; Cer, ceramide; DG, diacylglyceride; FA, fatty acid; LysoPC, lysoglycerophosphocholine; LysoPG, lysoglycerophosphoglycerol; PA, phosphatidic acid; PC, glycerophosphocholine; PE, glycerophosphoethanolamine; PG, glycerophosphoglycerol; PI, glycerophosphoinositol; PS, glycerophosphoserine; TG, triacylglyceride.

Using the “Lipid + data” where metabolite annotation was available, we assessed whether a small panel of metabolite features could differentiate between breeds. Features of the ten most statistically significant annotated metabolites from each pairwise *post hoc* analysis (lipids + and lipids –) were visualized as an average for each breed in a heatmap (Figure 3); with duplicate entries for the same lipid removed prior to plotting the heatmap. For all three sample types, the ability to discriminate between one breed and other breeds was feasible with a small number of metabolites. The results demonstrate that differences in the whole blood metabolome processed as DBS samples can be applied to study metabolic differences in biological studies with similar confidence as plasma. Data from a few metabolites may be representative of a breed, for example, acetyl carnitine (AcCa_1) and glycerophosphocholine (PC_1) are higher in Labrador retriever (Figure 3B). The prevalence of ceramides and lysoglycerophosphocholine in the DBS samples compared to the plasma samples may indicate the importance of cell membrane lipids in discriminating between breeds.

The ability of metabolite fingerprinting to identify biomarkers can be tested by providing samples blinded to analysts. In this study, two additional blinded DBS samples were used in an *ad hoc* test for the predictive capability of the PLS-DA models. Visual inspection from the PCA (Figure 1) had indicated that neither was likely to be cat. The PLS-DA of dog breeds identified the samples as unlikely to be Norfolk Terrier (Figure 2), and both were more consistent with Beagle (within the Beagle ellipses). Evaluating the blinded samples from their predicted values in the PLS-DA confirmed this visual inspection, with 15 of 16 identifying both as Beagle (Table 2), with similar predicted values for the two DBS volumes for each analysis. In one example where Labrador retriever had the highest score (lipids + analysis for “Blind 1” DBS 20), it had similar predicted values for Beagle.

**Table 2:** Breed prediction for the two blinded dried blood spot (DBS) samples based on PLS-DA predicted values.

**Analysis**	**Sample**	**Volume**	**Beagle**	**Labrador**	**Norfolk**	**PBGV**
HILIC –	Blind 1	DBS (20)	**0.52**	0.10	0.08	0.29
		DBS (40)	**0.48**	0.26	0.21	0.05
	Blind 2	DBS (20)	**0.62**	0.05	0.05	0.28
		DBS (40)	**0.78**	0.09	−0.05	0.18
HILIC +	Blind 1	DBS (20)	**0.41**	0.31	0.20	0.08
		DBS (40)	**0.40**	0.35	0.03	0.22
	Blind 2	DBS (20)	**0.77**	0.27	−0.05	0.01
		DBS (40)	**0.76**	0.22	−0.04	0.06
LIPIDS –	Blind 1	DBS (20)	**0.47**	0.24	0.13	0.16
		DBS (40)	**0.47**	0.09	0.14	0.30
	Blind 2	DBS (20)	**0.65**	0.11	−0.02	0.26
		DBS (40)	**0.64**	0.15	−0.07	0.27
LIPIDS +	Blind 1	DBS (20)	0.37	**0.39**	0.15	0.10
		DBS (40)	**0.37**	0.33	0.08	0.23
	Blind 2	DBS (20)	**0.53**	0.37	−0.18	0.28
		DBS (40)	**0.61**	0.23	−0.14	0.30

*The value highlighted in bold indicates the breed that the model predicts the blind sample to be*.

## Discussion

Large-scale biobanking initiatives have demonstrated advanced insights into disease predisposition and progress, identified environmental exposure and lifestyle risks, and provided unforeseen benefits, i.e., in the human COVID-19 pandemic (30, 31). Veterinary science would benefit considerably from such initiatives (32, 33). A constraint is that of providing sufficient numbers of high-quality samples from a wide-enough clinical base and providing robust statistical power. Plasma and serum are both used as standard samples to isolate and assess metabolites in blood fluid, and protocols have been developed and validated to minimize variations associated with sample collection and processing. However, the preparation of these to provide viable samples for analysis requires resources not usually present in primary care clinics and additional trained staff time. Any method that might minimize resource costs and that could use remnant samples from clinics would be a major breakthrough in advancing biobanking potential.

DBS cards have been used to assay specific analytes (34–36), and other -omics (37–39) are also used in dogs to diagnose specific canine pathologies (40, 41). Metabolomics is uniquely positioned to detect differences in biologically complex samples, as it represents the consequence of all aspects of the “DNA-RNA-Protein-Enzymatic activity” canon, and reflects aspects of the environment, including the host microbiome, diet, pathology and medication. A challenge for metabolomics compared to some -omics approaches is that both biotic and abiotic activities can continue to alter the metabolome after sample collection if appropriate protocols are not applied, making it more prone to collection and processing variability. The evidence that DBS can both stop metabolism and stabilize metabolites relatively quickly (14) makes this approach a tantalizing breakthrough for advancing large-scale biobanking. The reported purpose of this study was to determine whether remnant blood samples collected in a manner that could be prepared for biobanking in clinics (and possibly at pet owner homes) could provide samples of sufficient quality to discriminate biological classes by metabolomics fingerprinting.

The unsupervised and supervised multivariate analyses provided visual evidence that DBS at both 20 and 40 μL volumes could provide sufficient quality data to discriminate between species and dog breed size. There were differences in the number of features detected for the two volumes, likely to be due to the metabolites at the lower end of the level of detection, which had a subsequent effect on those best able to discriminate between breeds. Processes to control blood volume application and ways to normalize for volume differences, therefore, need to be considered. However, it may also be acceptable to rely on robust data detectable in all samples irrespective of volume, especially if using methods to normalize for different collected volumes (42–44). Both DBS volumes showed similar abilities to distinguish biological classes compared to the plasma samples produced using standard laboratory protocols. Furthermore, all were successful in indicating that both blinded samples were from Beagle dogs. Differences in metabolite features selected were observed between plasma and DBS, indicativ e of the fact that DBSs contain blood-borne cells with different metabolite composition and functions. From a biomarker perspective, this may be considered a confounder, as it may mask biomarkers in plasma transported from other sites in the body. However, cellular metabolites may also reflect a disease status (i.e., energy metabolism disorders or modifications in cellular lipid composition) and as DBSs also contain plasma-derived metabolites, those derived from other sites could still be sufficiently strong signals to discriminate between phenotype class. From our perspective, both plasma and DBSs are valuable samples for biobanking, providing data from both extracellular (plasma) and extracellular/intracellular (DBS) sources.

Metabolic fingerprinting provides a wide coverage of the metabolome when multiple UHPLC-MS assays are applied, making it a suitable method to discover biomarkers, and future biobank-based studies seeking early stage biomarkers in DBS should consider this approach. However, challenges to metabolite fingerprinting include delivering a practical diagnostic application and providing biological insights from biomarkers. There is a significant need to move away from fully untargeted metabolomic studies where derivation of metabolite structures from data collected can be a significant hurdle. The use of semi-targeted assays where metabolite chemical structures and, therefore, biological relevance are known prior to data collection ensures a simpler process to derive biological insights or identify biomarkers.

In conclusion, we have demonstrated that DBSs collected and processed in a way achievable in most veterinary clinics is a suitable sample for biomarker discovery, with similar diagnostic potential as plasma/serum, when metabolite fingerprinting is performed. Advantages of DBSs include minimal blood volume and processing, and given appropriate consent, the volumes tested (20 and 40 μL) make the acquisition of remnant blood from blood samples drawn for other reasons available for biobanking. The method also makes accessible high-quality samples for metabolomics analysis without the need for significant laboratory equipment resources (this may be especially relevant to veterinary and “in-field” sampling) and at a relatively low cost for sampling/storage. In principle, these features make biobanking of all clinical blood samples possible, thereby providing large-scale biobanking for cats and dogs, accelerating biomarker discovery of common diseases, and making the identification of rare diseases significantly more achievable.

## Data Availability Statement

The original contributions presented in the study are included in the article/Supplementary Material, further inquiries can be directed to the corresponding author/s.

## Ethics Statement

The animal study was reviewed and approved by WALTHAM Animal Welfare and Ethical Review Body.

## Author Contributions

DA, JA, LC-M, and WD contributed to the conception and design of the study. RR, CW, and AS conducted the research. LC-M, RW, GL, AS, and WD performed raw data processing and/or statistical analyses. DA wrote the first draft of the manuscript. WD wrote sections of the manuscript. All authors contributed to manuscript revision, read, and approved the submitted version.

## Funding

The work was funded by Mars Petcare UK and the funders had no role in the design, analysis or writing of this article. This work was also supported by Phenome Centre Birmingham (MR/M009157/1).

## Conflict of Interest

DA, JA, LC-M, and RR are employed by Mars Petcare. The remaining authors declare that the research was conducted in the absence of any commercial or financial relationships that could be construed as a potential conflict of interest.

## Publisher's Note

All claims expressed in this article are solely those of the authors and do not necessarily represent those of their affiliated organizations, or those of the publisher, the editors and the reviewers. Any product that may be evaluated in this article, or claim that may be made by its manufacturer, is not guaranteed or endorsed by the publisher.
