# Supplementary material for: Suitability of Dried Blood Spots for Accelerating Veterinary Biobank Collections and Identifying Metabolomics Biomarkers With Minimal Resources
Source: Front Vet Sci. 2022 Jun 22;9:887163. doi: 10.3389/fvets.2022.887163 (PMC9258959; doi:10.3389/fvets.2022.887163)
Supplement: Supplementary file 1 [file Data_Sheet_1.docx]

**Supplementary Information**

**1. UHPLC-MS assays**

### *Materials:* Acetonitrile (ACN), methanol (MeOH), water (H_2_O), and isopropanol (propan-2-ol, IPA)

### were all LC-MS grade (Optima brand, Fisher Scientific).

*HILIC assay:* All samples were analyzed applying a Dionex UltiMate 3000 UHPLC system coupled with a heated electrospray Q Exactive Focus mass spectrometer (Thermo Fisher Scientific). HILIC assays used an Accucore150-Amide-HILIC column (100 x 2.1mm, 2.6μm, ThermoFisher Scientific). For positive ion analysis, mobile phase A was 10 mM ammonium formate dissolved in acetonitrile/water/formic acid (95:4.9:0.1 (v/v)) and mobile phase B was 10 mM ammonium formate dissolved in acetonitrile/water/formic acid (50/49.9/0.1 (v/v)). For negative ion analysis, mobile phase A was 10 mM ammonium acetate dissolved in acetonitrile/water/acetic acid (95:4.9:0.1 (v/v)) and mobile phase B was 10 mM ammonium acetate dissolved in acetonitrile/water/acetic acid (50/49.9/0.1 (v/v)). The gradient elution applied for positive and negative ion mode was t=0.0, 1% B; t=1.0, 1% B; t=3.0, 15% B; t=6.0, 50% B; t=9.0, 95% B; t=10.0, 95% B; t=10.5, 1% B; t=14.0, 1% B. All changes were linear (curve = 5) and the flow rate was 0.50 mL.min^-1^. Column temperature was 35°C and injection volume was 2μL. Data were acquired in positive and negative ionisation modes separately (70 – 1050 *m/z*) with a mass resolution 70,000 (FWHM, *m/z* 200). Ion source parameters applied were Sheath gas = 55 arbitrary units, Aux gas = 35 arbitrary units, Sweep gas = 4 arbitrary units, Spray Voltage = 3.2kV (positive ion) and 2.7kV (negative ion), Capillary temp. = 380°C, Aux gas heater temp. = 440°C. Thermo Exactive Tune (2.8 SP1, build 2806) software controlled the instruments and data acquisition. All data were acquired in profile mode. All data were collected as MS1 data in profile mode with the exception of four QC sample injections (injections 6-9) where MS/MS data were collected in the “Discovery mode” setting over different precursor *m/z* ranges (70−210 *m/z*; 200−310 *m/z*; 300−410 *m/z*; 400-510 *m/z*; 500-1050 *m/z*) using stepped normalized collision energies (positive ion mode: 20, 40, 100%; negative ion mode: 40, 60, 130%). All samples were maintained at a temperature of 4°C in the autosampler. All samples derived from the 20μL DBS study were analysed first applying both assays in positive ion mode followed by negative ion mode (sample analysis was completed in <2 days after sample reconstitution/preparation). All samples derived from the 40μL study were analysed next applying both assays in positive ion mode followed by negative ion mode (sample analysis was completed in <2 days after sample reconstitution/preparation). All samples derived from the plasma study were analysed last applying both assays in positive ion mode followed by negative ion mode (sample analysis was completed in <2 days after sample reconstitution/preparation). No instrument maintenance was applied between different sample types or ion modes.

*Lipids assay:* All samples were analyzed applying a Dionex UltiMate 3000 UHPLC system coupled with a heated electrospray Q Exactive Focus mass spectrometer (Thermo Fisher Scientific). Lipid assays used a reversed-phase Hypersil GOLD C_18_ column (100 x 2.1 mm, 1.9μm; Thermo Fisher Scientific). Mobile phase A was 10 mM ammonium formate dissolved in acetonitrile/water/formic acid (60:39.9:0.1 (v/v)) and mobile phase B was 10 mM ammonium formate dissolved in isopropanol/acetonitrile/water/formic acid (85.5/9.5/4.9/0.1 (v/v)). The gradient elution applied was t=0.0, 20% B; t=0.5, 20% B, t=8.5, 100% B; t=9.5, 100% B; t=11.5, 20% B; t=14.0, 20% B. All changes were linear (curve = 5) and the flow rate was 0.40 mL/min. Column temperature was 55 °C and injection volume was 2μL. Data were acquired in positive and negative ionisation mode separately (150 –2000 *m/z*) with a mass resolution 70,000 (FWHM, *m/z* 200). Ion source parameters applied were Sheath gas = 48 arbitrary units, Aux gas = 15 arbitrary units, Sweep gas = 0 arbitrary units, Spray Voltage = 3.2kV (positive ion) / 2.7kV (negative ion), Capillary temp. = 380°C, Aux gas heater temp. = 450°C. Thermo Exactive Tune (2.8 SP1, build 2806) software controlled the instruments and data acquisition. All data were acquired in profile mode. All data were collected as MS1 data in profile mode with the exception of four QC sample injections (injections 6-9) where MS/MS data were collected in the “Discovery mode” setting over different precursor *m/z* ranges (HILIC: 70−210 *m/z*; 200−310 *m/z*; 300−410 *m/z*; 400-510 *m/z*; 500-1050 *m/z*; Lipids: 150−510 *m/z*; 500−710 *m/z*; 700−860 *m/z*; 850−1010 *m/z*; 1000−2000 *m/z*) using stepped normalized collision energies (HILIC and lipids positive ion mode: 20, 40, 100%; HILIC and lipids negative ion mode: 40, 60, 130%). All samples were maintained at a temperature of 4°C in the autosampler. All samples derived from the 20μL study were analysed first applying both assays in positive ion mode followed by negative ion mode (sample analysis was completed in <2 days after sample reconstitution/preparation). All samples derived from the 40μL study were analysed next applying both assays in positive ion mode followed by negative ion mode (sample analysis was completed in <2 days after sample reconstitution/preparation). All samples derived from the plasma study were analysed last applying both assays in positive ion mode followed by negative ion mode (sample analysis was completed in <2 days after sample reconstitution/preparation). No instrument maintenance was applied between different sample types or ion modes.

**2. Raw data processing**

*Raw data processing.* Vendor format raw data files (.RAW) were converted to the mzML file format using the open-source ProteoWizard software (Chambers *et al*., 2012). Deconvolution was performed by the R package XCMS (version 3.6.1 running in the Galaxy environment) (Smith *et al*., 2006). The R package IPO (Libiseller *et al*., 2015) was used to optimise and obtain XCMS peak picking parameters for each of the different sample types (DBS-20µL, DBS-40 µL and plasma-40 µL are reported in this order below) and assays: min peak width (3; all assays); max peak width (30; all assays); ppm (11.5, 13.5, 14.5 for HILIC positive; 11.2, 10, 15.3 for HILIC negative; 12, 12.5, 9.5 for lipid positive; 12, 12, 12.5 for lipid negative); mzdiff (0.0133, 0.00835, 0.0034 for HILIC positive; 0.00395, 0.0034, 0.00395 for HILIC negative; 0.00615, 0.00725, 0.00395 for lipid positive; 0.00725, 0.00725, 0.00725 for lipid negative); bw (0.25); mzwid (0.00835, 0.00835, 0.01815 for HILIC positive; 0.00345, 0.02795, 0.0059 for HILIC negative; 0.01325, 0.0157, 0.01325 for lipid positive; 0.0353, 0.03285, 0.0108 for lipid negative); minfrac (0.5). A data matrix of metabolite features (i.e. *m/z*-retention time pairs) versus samples was constructed for each sample type and assay. Twelve different data matrices were constructed for three sample types (DBS-20µL, DBS-40µL, plasma-40µL) and four assays (HILIC-negative, HILIC-positive, Lipids-negative, Lipids-positive).

**References**

Chambers *et al*., 2012. A cross-platform toolkit for mass spectrometry and proteomics. *Nature biotechnology*, *30*(10):918-920

Libiseller *et al*., 2015. IPO: a tool for automated optimization of XCMS parameters. *BMC bioinformatics*, *16*(1):1-10

Smith *et al*., 2006. XCMS: processing mass spectrometry data for metabolite profiling using nonlinear peak alignment, matching, and identification. *Analytical Chemistry*, *78*(3):779-787

**3. PCA scores plots: All samples**

**A.** PCA scores plots (PC1 vs PC2) for data collected for three sample types applying the HILIC negative ion mode assay. The sample classes are pooled QC:yellow; domestic shorthair cat:green, Norfolk terrier:light blue, Labrador retriever:dark blue, Beagle:red and Petit Basset Griffon Vendeen:pink. The percentage variance displayed for PC1 and PC2 are 28.8% and 6.2% for DBS-20µL, 24.8% and 6.1% and 7.9% for DBS-40µL and 27.4% and 7.9% for plasma.


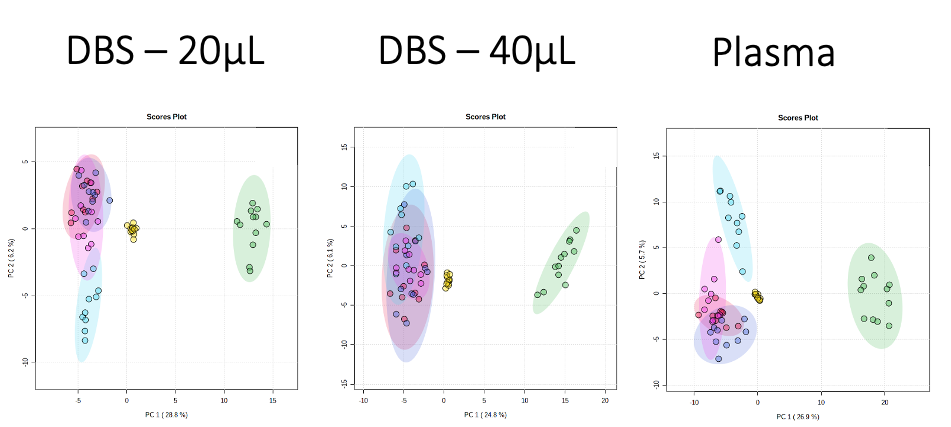


**B.** PCA scores plots (PC1 vs PC2) for data collected for three sample types applying the Lipids negative ion mode assay. The sample classes are yellow (pooled QC samples), green (domestic shorthair cat), light blue (Norfolk terrier), dark blue (Labrador retriever) and pink (Petit Basset Griffon Vendeen). The percentage variance displayed for PC1 and PC2 are 31.4% and 8.8% for DBS-20µL, 38.2% and 9.7% for DBS-40µL and 30.2% and 11.5% for plasma.


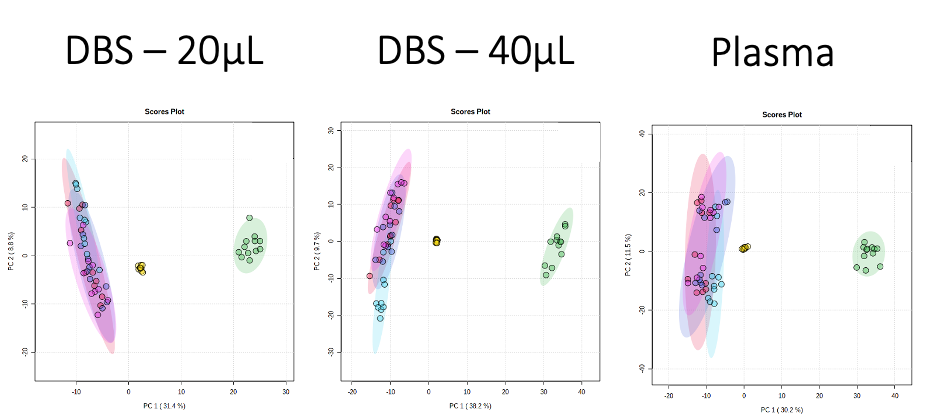


**C.** PCA scores plots (PC1 vs PC2) for data collected for three sample types applying the Lipids positive ion mode assay. The sample classes are yellow (pooled QC samples), green (domestic shorthair cat), light blue (Norfolk terrier), dark blue (Labrador retriever) and pink (Petit Basset Griffon Vendeen). The percentage variance displayed for PC1 and PC2 are 31.1% and 8.2% for DBS-20µL, 37.2% and 11.4% for DBS-40µL and 33.2% and 13.2% for plasma.


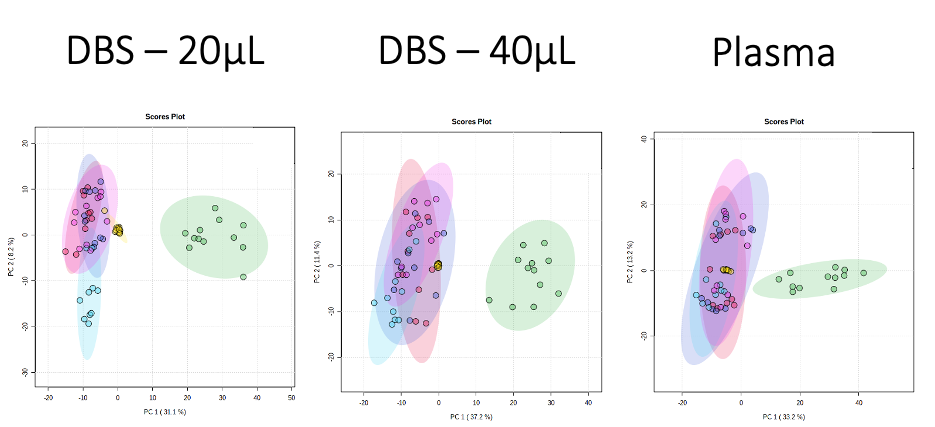


**4. PLS-DA scores plots**

**A.** PLS-DA scores plots for data collected for three sample types applying the HILIC negative ion mode assay. The sample classes are Norfolk terrier:yellow), Labrador retriever:turquoise, Beagle:dark blue, Petit Basset Griffon Vendeen:purple, Blind sample 1:Orange; Blind sample 2:Green. The mean error rates across 5-fold cross-validation models repeated 20 times for DBS 20, DBS 40 and Plasma were 0.23, 0.23 and 0.33, respectively. The component 1 and component 2 variances were 30 and 10% (DBS - 20µL), 31 and 11% (DBS - 40µL), 8 and 8% (plasma).

**
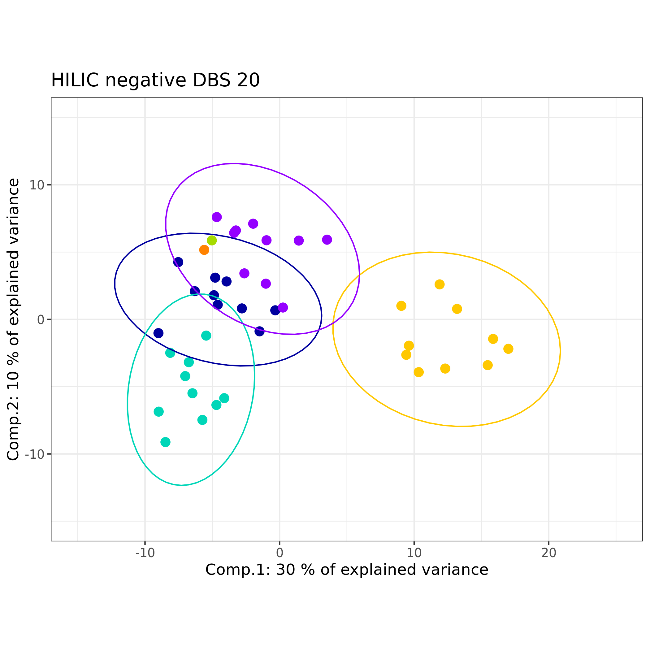

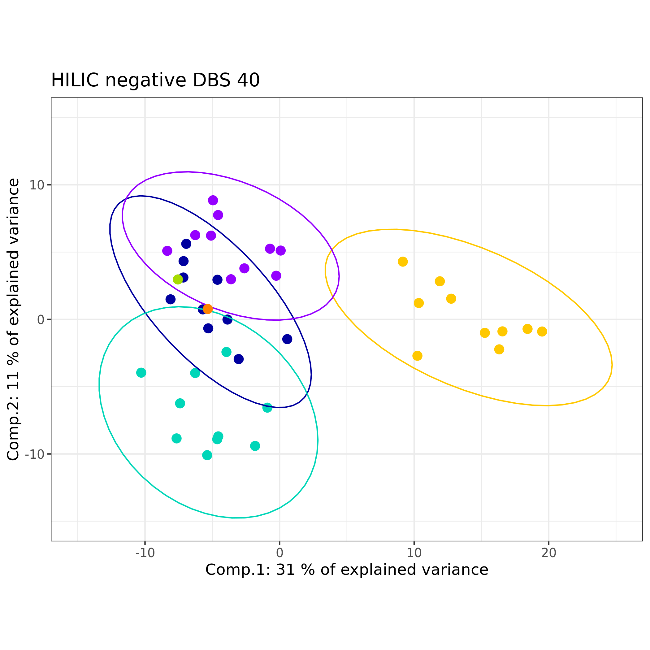

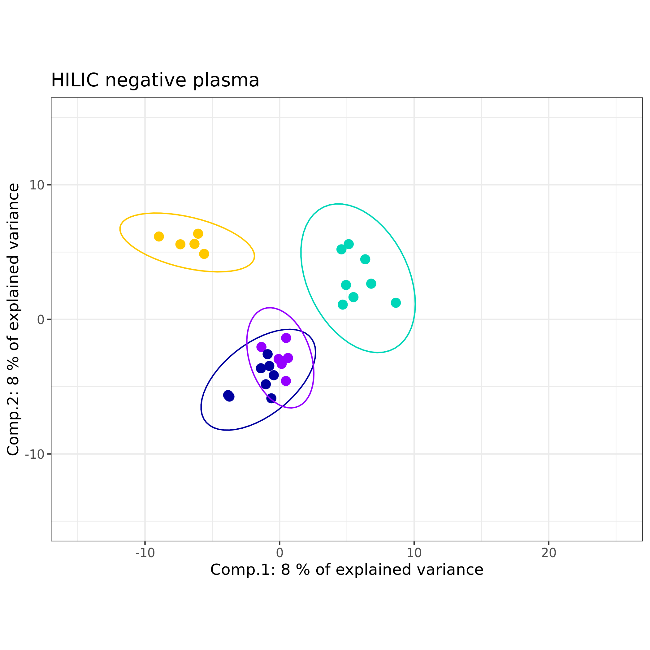
**

**B.** PLS-DA scores plots for data collected for three sample types applying the Lipids negative ion mode assay. The sample classes are Norfolk terrier:yellow), Labrador retriever:turquoise, Beagle:dark blue, Petit Basset Griffon Vendeen:purple, Blind sample 1:Orange; Blind sample 2:Green. The mean error rates across 5-fold cross-validation models repeated 20 times for DBS 20, DBS 40 and Plasma were 0.28, 0.27 and 0.25, respectively. The component 1 and component 2 variances were 38 and 10% (DBS - 20µL), 42 and 11% (DBS - 40µL), 34 and 11% (plasma).

**
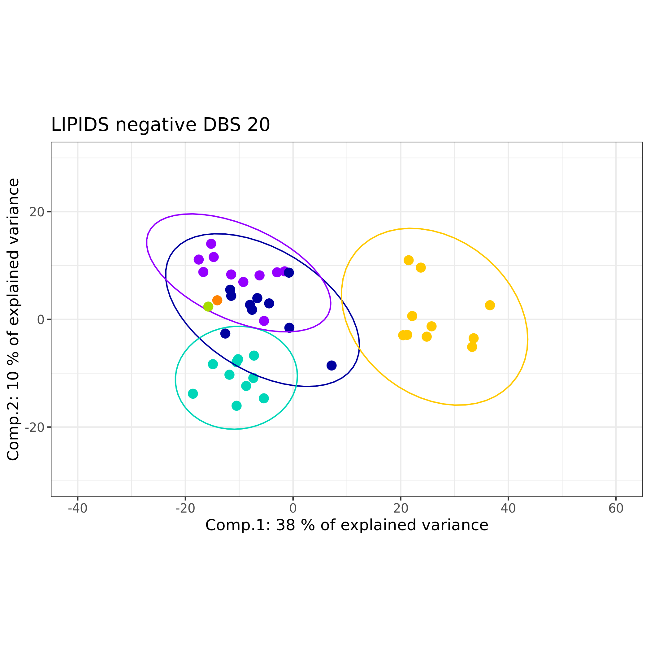

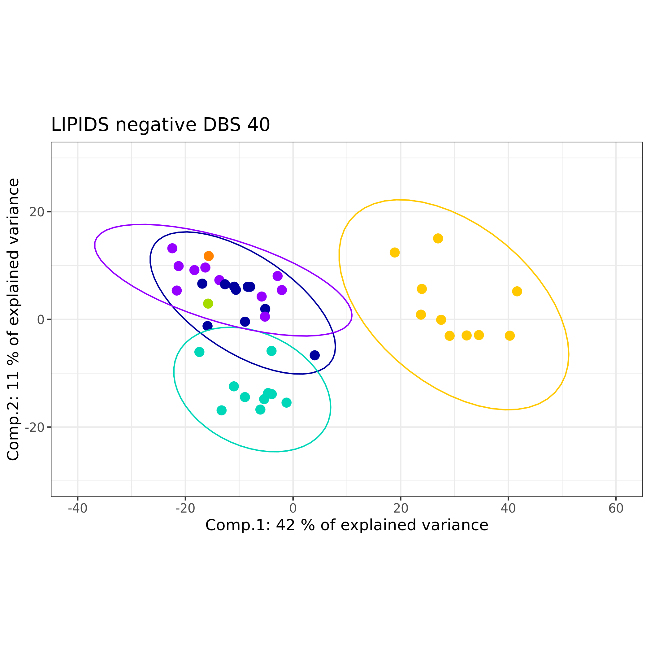

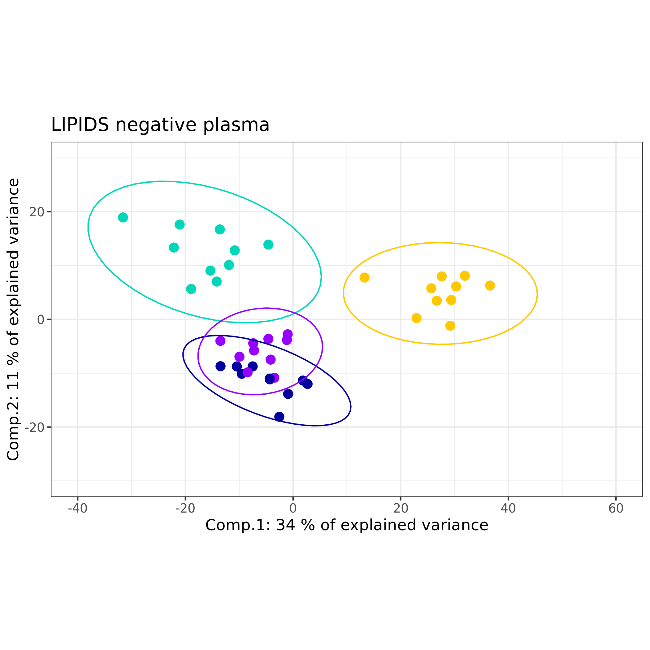
**

**C.** PLS-DA scores plots for data collected for three sample types applying the Lipids positive ion mode assay. The sample classes are Norfolk terrier:yellow), Labrador retriever:turquoise, Beagle:dark blue, Petit Basset Griffon Vendeen:purple, Blind sample 1:Orange; Blind sample 2:Green. The mean error rates across 5-fold cross-validation models repeated 20 times for DBS 20, DBS 40 and Plasma were 0.34, 0.32 and 0.26, respectively. The component 1 and component 2 variances were 40 and 9% (DBS - 20µL), 43 and 11% (DBS - 40µL), 34 and 8% (plasma).

**
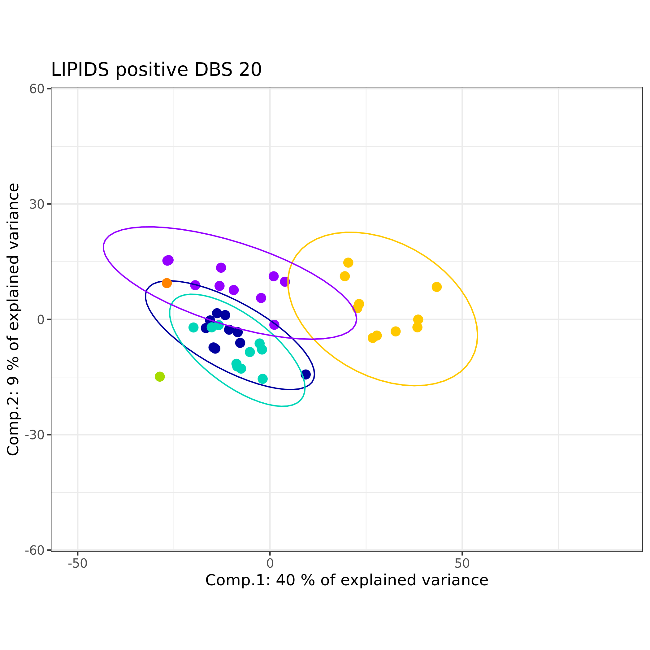

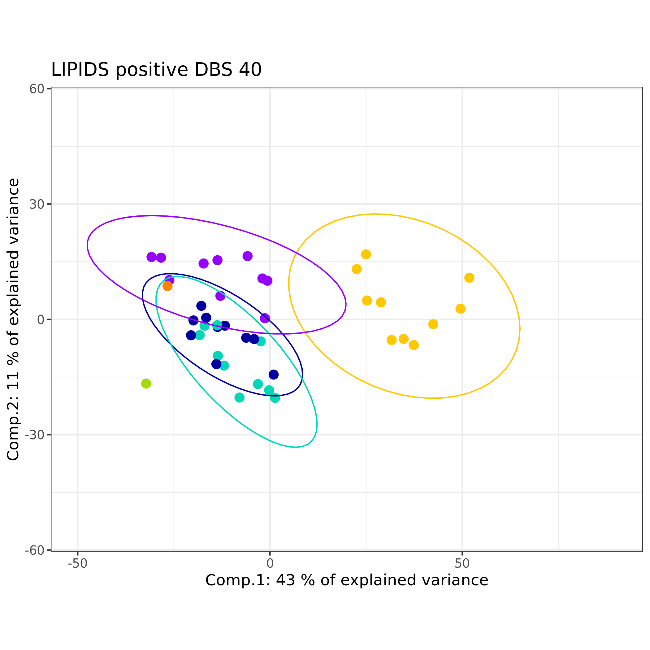

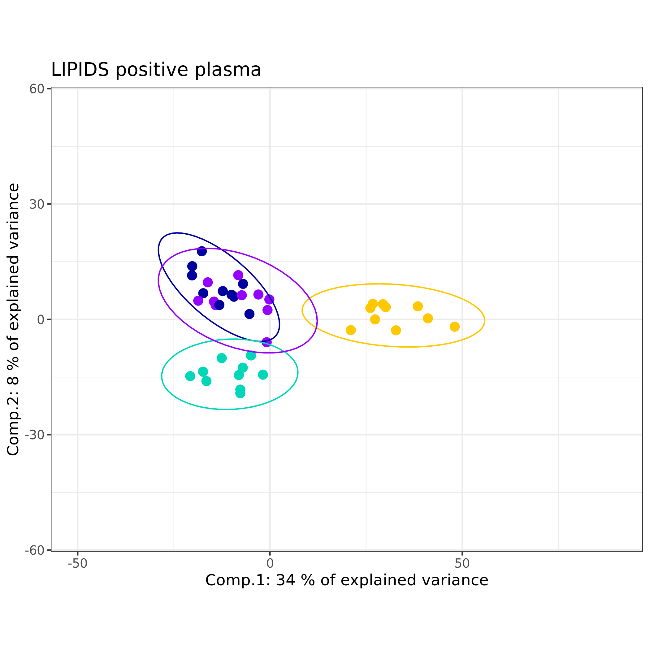
**
